# Carcinome épidermoïde du sein: à propos d’un cas et revue de la literature

**DOI:** 10.11604/pamj.2016.24.213.6120

**Published:** 2016-07-12

**Authors:** Mounia Ziyadi, Majdouline Boujoual, Hanane Raiteb, My Abdellah Babahabib, Jawad Kouach, Driss Rahali Moussaoui, Mohammed Dehayni

**Affiliations:** 1 Service de Gynécologie Obstétrique, Hôpital Militaire Instructif Mohammed V Rabat, Maroc; 2 Service de Gynécologie Obstétrique, HMIMV Rabat, Faculté de Médecine Tanger, Maroc

**Keywords:** Carcinome épidermoïde, sein, diagnostic, traitement, pronostic, Squamous cell carcinoma, breast, diagnosis, treatment, prognosis

## Abstract

Le carcinome épidermoïde du sein est une tumeur rare, d'origine métaplasique l'ethiopathogénie est controversé, le diagnostic est histologique après avoir éliminé l'origine métastatique, la clinique et la radiologie ne sont pas spécifique le traitement rejoint celui du carcinome canalaire infiltrant, le pronostic est péjoratif liée à la taille tumorale et à l'envahissement ganglionnaire, on rapporte un cas de carcinome épidermoïde du sein chez une patiente de 39 ans ayant consulté au service de gynécologie obstétrique de hôpital militaire de Rabat, et à travers cette observation on va mettre le point sur les différentes caractéristiques de cette entité qui reste rare.

## Abstract (en)

Squamous cell carcinoma of the breast is a rare tumour of metaplasic origin; etiopathogeny is controversial; diagnosis is based on histological examination after eliminating the primary tumor; clinical and radiological examination is not specific; treatment is the same as for invasive ductal carcinoma; prognisis is poor due to tumor size and lymph node involvement. We report a case of breast squamous cell carcinoma in a 39 years old patient who was examined in the obstetrics and gynecology department of the military hospital in Rabat. Patient observation allowed us to specify the clinical characteristics of this entity which remains rare.

## Introduction

Le carcinome épidermoïde ou carcinome à cellules squameuses du sein est une tumeur métaplasique rare dont l'histogenèse est controversée. Elle se caractérise par une évolution rapide et par un traitement non codifié. Nous rapportons un nouveau cas de carcinome épidermoïde du sein, nous insisterons à travers une revue de littérature sur les caractéristiques de cette forme particulière de cancer du sein.

## Patient et observation

**Figure 1:**
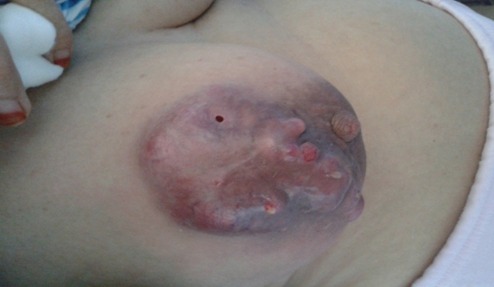
Aspect macroscopique de la tumeur: masse péri aréolaire du sein droit étendue aux quadrants externes d’aspect bourgeonnant, inflammatoire et surinfecté faisant 14 cm /11 cm

**Figure 2:**
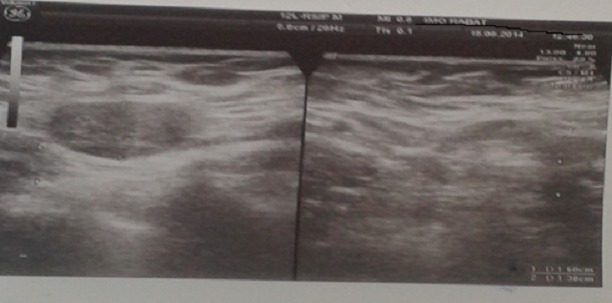
Aspect échographique montrant une formation hypoéchogène hétérogène de contours irréguliers renfermant des zones kystiques

**Figure 3:**
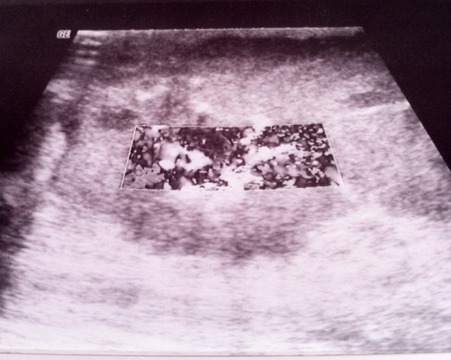
La masse est hypervascularisée au Doppler

Patiente âgée de 49 ans, mère de quatre enfants, ayant comme antécédent une tumorectomie du sein droit dont l'étude anatomo-pathologique a objectivé une mastopathie fibrokystique, qui a présenté depuis 6 mois une énorme masse du sein droit augmentant rapidement de volume avec mastodynies. Son examen clinique a retrouvé une masse péri aréolaire du sein droit, s'étendant aux quadrants externes, mesurant 14 cm /11 cm, d'aspect bourgeonnant, inflammatoire, suintant, surinfecté, rénitente à la palpation et adhérente au plan superficiel avec rétraction mamelonnaire (Figure 1). L'examen des aires ganglionnaires a retrouvé deux adénopathies axillaires mesurant 2 cm, mobiles et douloureuses. L'échographie mammaire a mis en évidence une formation hypoéchogène hétérogène de contours irréguliers renfermant des zones kystiques et prenant fortement le doppler couleur mesurant 6,1 x 4,25 cm, associée à de multiples adénopathies axillaires dont la plus volumineuse mesurait 1,6 x 1,4 cm (Figure 2, Figure 3). Une micro biopsie a été alors réalisée révélant un carcinome infiltrant de haut grade avec différenciation épidermoïde. Son bilan d'extension n'a pas révélé de localisation secondaire. La patiente a bénéficié alors d'un Patey droit. Les suites post opératoires ont été simples. L'examen anatomo pathologique a confirmé le diagnostic de carcinome épidermoïde du sein droit, grade III SBR, mesurant 10 cm, caractérisé par la présence de nécrose et d'emboles vasculaires avec des Récepteurs Hormonaux et Hercept test négatif. Un ganglion lymphatique était envahi sur 13 ganglions prélevés. La patiente a été adressée au service d'oncologie pour un traitement adjuvant.

## Discussion

Les carcinomes épidermoïdes primitifs du sein sont rares, représentent 0,1 à 2% de l'ensemble des carcinomes du sein et appartiennent au groupe hétérogène des carcinomes métaplasiques mammaires [1]. Selon l´OMS [2], le carcinome épidermoïde du sein fait partie des carcinomes canalaires infiltrants comportant des remaniements métaplasiques de type épidermoïde, en l´absence de toute autre composante néoplasique ductale ou mésenchymateuse et d'autre foyer de carcinome épidermoïde à distance [3]. Ces tumeurs naissent par métaplasie totale ou partielle, transformant une cellule épithéliale, myoépithéliale ou totipotente de réserve en un autre type de cellule épithéliale ou mésenchymateuse [3]. D'autre part, elles pourraient survenir à partir d'un kyste dermoïde mammaire, d´un abcès chronique du sein, ou à partir d´un cystosarcome phyllode [3]. Le carcinome épidermoïde du sein atteint la femme entre 30 et 80 ans avec prédominance à l'âge de 55 ans [2]. Cliniquement, il se présente sous forme d'une masse mammaire dont la taille moyenne est de 5 cm avec des extrêmes de 2 à 16 cm. Par ailleurs, les tumeurs de grande taille tendent à subir une dégénérescence kystique centrale avec envahissement et ulcération de la peau en regard, ce qui rend parfois difficile la distinction entre carcinome épidermoïde mammaire primitif et secondaire [3, 4]. Radiologiquement, l'aspect n'est pas spécifique. Il s'agit généralement d'une masse arrondie, sans spicule, partiellement irrégulière à centre nécrotique ou kystique ce qui explique l'aspect pseudo kystique ou abcédé que l'on retrouve [2, 5, 6]. Le diagnostic préopératoire peut être réalisé par simple aspiration cytologique ou par une drill-biopsie comme c´est le cas de notre patiente [2]. Toutefois, l´examen histopathologique est indispensable pour rechercher une éventuelle composante adénoïde et éliminer une éventuelle extension locale d'un carcinome épidermoïde de la peau en regard, du mamelon ou d'une métastase à distance. L'immunohistochimie montre une expression des cellules tumorales épithéliales des cytokératines de haut poids moléculaire notamment les CK14, CK5/6 et CK17 [7]. Toutefois, la majorité de ces tumeurs n'expriment pas les récepteurs hormonaux, de même que l'amplification de l'Her 2, ce qui concorde avec notre patiente. En revanche le caractère prolifératif (Ki 67) a été fortement démontré par l'étude menée par grenier et al [8]. Le traitement est similaire à celui des carcinomes canalaires infiltrants du sein de même taille et au même stade d´évolution, il comporte habituellement une mastectomie avec curage ganglionnaire axillaire suivie d´une radiothérapie et d´une chimiothérapie [2]. En effet, la chimiothérapie néo adjuvante n'est pas justifiée pour envisager un traitement conservateur puisque ses résultats sont médiocres [9]. De même, l'hormonothérapie a peu de place compte tenu de l'absence de surexpression des récepteurs hormonaux dans cette forme histologique. Les principaux facteurs pronostiques représentent : la taille tumorale, l'envahissement ganglionnaire axillaire [10], la composante fusiforme, la nécrose, et l'acantholyse cellulaire [10]. Le pronostic des carcinomes épidermoïdes reste péjoratif avec une survie moyenne à 5 ans estimée entre 50 et 63% [9, 10]. Les perspectives d'avenir seraient les thérapies ciblées, notamment du récepteur EGFR, pour améliorer le pronostic [10].

## Conclusion

Le carcinome épidermoïde du sein est une tumeur métaplasique rare ayant des caractéristiques cliniques et radiologiques aspécifiques, et dont le pronostic reste péjoratif, soulignant l’intérêt des études permettant une meilleure connaissance de leur histogénèse et une prédiction de leur profil évolutif afin de mieux codifier la prise en charge.
